# Trait-specific Selection Signature Detection Reveals Novel Loci of Meat Quality in Large White Pigs

**DOI:** 10.3389/fgene.2021.761252

**Published:** 2021-11-16

**Authors:** Yu Shen, Haiyan Wang, Jiahao Xie, Zixuan Wang, Yunlong Ma

**Affiliations:** Key Laboratory of Agricultural Animal Genetics, Breeding, and Reproduction of the Ministry of Education and Key Laboratory of Swine Genetics and Breeding of the Ministry of Agriculture, Huazhong Agricultural University, Wuhan, China

**Keywords:** selective sweeps, phenotypic gradient differential population pairs, population differentiation-based methods, functional annotation, meat quality

## Abstract

In past decades, meat quality traits have been shaped by human-driven selection in the process of genetic improvement programs. Exploring the potential genetic basis of artificial selection and mapping functional candidate genes for economic traits are of great significance in genetic improvement of pigs. In this study, we focus on investigating the genetic basis of five meat quality traits, including intramuscular fat content (IMF), drip loss, water binding capacity, pH at 45 min (pH45min), and ultimate pH (pH24h). Through making phenotypic gradient differential population pairs, Wright’s fixation index (F_ST_) and the cross-population extended haplotype homozogysity (XPEHH) were applied to detect selection signatures for these five traits. Finally, a total of 427 and 307 trait-specific selection signatures were revealed by F_ST_ and XPEHH, respectively. Further bioinformatics analysis indicates that some genes, such as *USF1*, *NDUFS2*, *PIGM*, *IGSF8*, *CASQ1*, and *ACBD6*, overlapping with the trait-specific selection signatures are responsible for the phenotypes including fat metabolism and muscle development. Among them, a series of promising trait-specific selection signatures that were detected in the high IMF subpopulation are located in the region of 93544042-95179724bp on SSC4, and the genes harboring in this region are all related to lipids and muscle development. Overall, these candidate genes of meat quality traits identified in this analysis may provide some fundamental information for further exploring the genetic basis of this complex trait.

## Introduction

The most important purpose of pig breeding is the genetic improvement of important economic traits (Price 1999; Zhang et al., 2020). In past decades, the aim of genetic improvement of pig breeding has mainly focused on improving meat production through growth rate and feed efficiency, lean percentage, and decreasing backfat thickness. As expected, the genetic gain of these traits is successful in most selection programs. Simultaneously, human-driven selection has also indirectly shaped the meat quality traits, such as intramuscular fat content (IMF), pH values, drip loss (DL), and meat color (Herault et al., 2018). From the perspective of population genetics, the effect of human-driven selection as well as natural selection would leave detectable signatures in the genome. Therefore, detecting the selection signatures of these important economic traits can provide an insight into molecular mechanisms by which genomic fragments shape phenotypic diversity (Qanbari and Simianer 2014).

Although genomic selection has been widely applied in animal breeding in recent years, it is still difficult to carry out genetic improvement of meat quality traits in pigs (Zhan et al., 2021). The most critical factor is the high cost of measuring meat quality traits, which makes it difficult to build a large enough reference population. Therefore, marker-assisted selection (MAS) based on functional candidate genes is still an important choice for genetic improvement of meat quality traits. So far, genome-wide association analysis (GWAS) has been conducted to reveal some functional candidate genes, such as *PRKAG3*, *MC4R*, and *PIT1*, which have been identified in different populations to be related to meat quality traits (Yu et al., 1999; Milan et al., 2000; Lopez et al., 2015; Zhang et al., 2019). In addition, there are a lot of quantitative trait loci (QTL) associated with meat quality in pig QTLdb, more specifically, 1092 QTLs are associated with drip loss, 851 QTLs are associated with intramuscular fat content, and 667 QTLs are associated with meat color (http://www.animalgenome.org/QTLdb/SS/index) (Hu et al., 2019). However, it is still a challenge to reveal the exact genetic mechanism of meat quality traits.

To further explore the genetic mechanism of meat quality traits, we put forward a hypothesis: although the allele frequency has increased underlying human-driven selection, the loci related to meat quality traits are still polymorphic (Ma et al., 2019). Here, we construct three phenotypic gradient differential population pairs based on phenotype and use population differentiation–based methods to detect selection signatures associated with meat quality traits. If successful, the results can provide more information for understanding the genetic mechanism of meat quality traits and facilitate the genetic improvement of meat quality traits through marker-assisted selection.

## Materials and Methods

### Animals and Phenotypes

In this study, a total of 233 castrated large white pigs were used. The experimental pigs were raised in the same farm, had a common diet, and drank water freely. The same standard management conditions were applied in the whole process of the experiment. Antibiotics are banned in the 3 months before slaughter. Finally, healthy individuals were chosen and slaughtered at around 90 Kg weight. The meat samples of longissimus lumborum from all pigs were collected for measuring meat quality traits, including IMF, DL, water binding capacity (WBC), pH45min, and pH24h (Horwitz and Latimer, 1995; Cherel et al., 2011; Zhan et al., 2021; Zhang et al., 2019).

Here, IMF was measured as percentage of lipid (lipid weight per 100 g of muscle tissue). Correspondingly, the Soxhlet extraction method was used following the standard AOAC Official method in foods (Horwitz and Latimer, 1995). The pH values of each sample were measured by a waterproof meat pH meter (Hanna, Romania). The electrode of the pH meter was calibrated in buffers at pH 7.00 and 4.00 before pH measurement. To calculate DL, we first measured the weight of the meat sample with 2.5 cm thickness at the 12th rib. Second, the final weight was measured, and after that, the meat sample was suspended in a sealed tube for 48 h at 4°C. Finally, the formula of (original weight—final weight)/original weight × 100 was applied to predict the DL. WBC was evaluated according to the Graua–Hamma method (Hamm, 1986). In this study, all five traits were measured in triplicate to reduce random error, and mean values were applied to the following analysis.

### Genotyping and Quality Control

In this study, genomic DNA was extracted from ear tissue using a standard phenol-chloroform method. All 233 castrated large white pigs were genotyped using Illumina PorcineSNP60 BeadChips, which includes 62,163 single nucleotide polymorphisms (SNPs). Then, quality control was performed using the following criteria: i) SNP missing rate <0.05, ii) individual call rate >0.90, iii) SNPs in Hardy–Weinberg equilibrium (*p* > 10e–6), iv) SNP minor allele frequency ˃0.05, v) autosomal SNPs with known positions extracted. After quality control, 11,624 SNPs with minimum allele frequency less than 0.05 were deleted, and 42 markers were deleted after the Hardy–Weinberg test (*p* ≤ 10e-6). Finally, the data set contained 37,061 autosome SNPs with an average inter-marker spacing of 62.06 kb. The genotype data can be downloaded from Figshare (https://figshare.com/s/cd815d8930c75561392c). The BEAGLE software was then applied to impute the missing genotypes and infer haplotypes (Browning and Browning 2016). The PLINK (Version 1.90) software was used to measure the linkage disequilibrium and allele frequency in large white pigs (Purcell et al., 2007). Principal component analysis (PCA) was further performed using the PLINK (Version 1.90) software. To visualize the LD decay, the r2 values for 1 kb distance bins were averaged and drawn using the R program.

### Detection of Trait-Specific Selection Signatures

To reveal trait-specific selection signatures, we construct three phenotypic gradient differential population pairs based on phenotype first and then identify selection signatures using population differentiation–based methods in this study. The detailed analysis flow applied the following steps: i) ranking by phenotypic values of each trait; ii) based on the rank of phenotypic values, we equally divided the source population into high and low phenotypic subpopulations and recorded them as first population pair; iii) based on step ii, we chose 75 individuals with a higher phenotype from the high phenotype subpopulation and 75 individuals with a lower phenotype from the low phenotype subpopulation to create the second population pair; iv) similarly, we further chose 45 individuals with a higher phenotype and 45 individuals with a lower phenotype from the second population pair subpopulations to create the third population pair; v) population differentiation–based methods XPEHH and F_ST_ were separately applied to identify selection signatures in three phenotypic gradient differential population pairs (Sabeti et al., 2007; Weir and Cockerham 1984).

In this analysis, the XPEHH scores do not need to be standardized. The empirical *p*-values were generated by genome-wide ranking of F_ST_ and XPEHH scores, respectively. The trait-specific selection signatures were defined using the following two criteria: i) the SNPs with *p*-value < .01 were considered as significant loci; ii) from first to second to third population pair, the F_ST_ or XPEHH scores of each significant locus displayed a gradient change.

### Functional Annotation for Trait-Specific Selection Signatures

To reveal the potential biological functions of trait-specific selection signatures, we defined the trait-specific selection region as the genomic region within a distance of 200 kb around the trait-specific selection signatures. The BioMart program in ensembl (https://www.ensembl.org/index.html) was employed to search the genes and their orthologous genes of mouse harboring the trait-specific selection signature regions. Then, the database of Mouse Genome Informatics (MGI) was used to perform functional annotation (Dickinson et al., 2016). The trait-specific selection signature regions were also annotated using pigQTLdb in this analysis. Based on the genes harboring in trait-specific selection signature regions, GO and pathway analysis was used for the functional annotation and classification using DAVID 6.8 (https://david.ncifcrf.gov/) (Huang et al., 2009). The GO terms and pathways with *p*-value < .05 were considered as significant after Bonferroni correction.

## Results

### Phenotypes Among Phenotypic Gradient Differential Population Pairs

**FIGURE 1:**
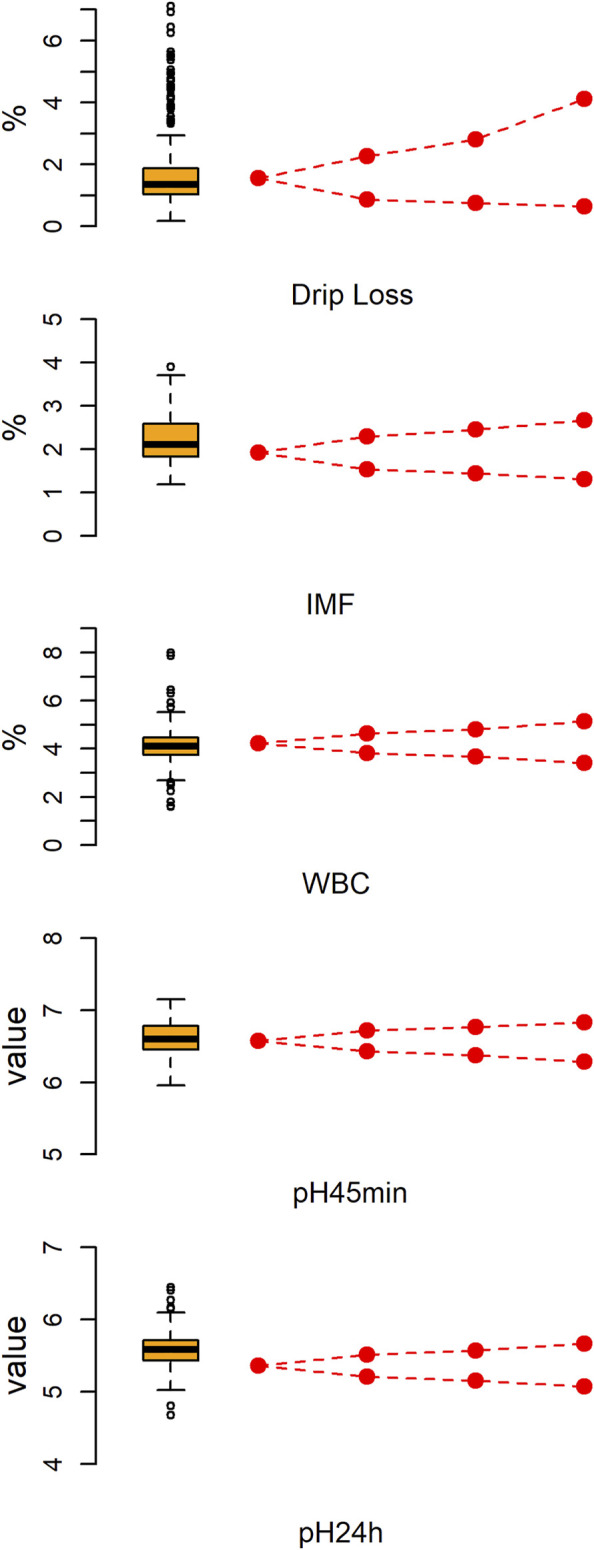
The phenotypic distribution of five meat quality traits and the tendency of phenotypic values in phenotypic gradient differential population pairs. The left is a box-whiskers plot. The right line graph displays a gradual change tendency of phenotypic mean value from the source population to first through third population pairs.

Figure 1 shows the descriptive statistics of five meat quality traits of large white pigs. As shown in Supplementary Table S1, there was a significantly positive association between DL and the other traits. However, IMF only has a significant positive correlation with WBC. For each trait, all 233 pigs were divided into two subpopulations: high and low phenotype value groups. Then, the extreme individuals were chosen to construct three phenotypic gradient differential population pairs based on the ranking of phenotype values. As expected, the average phenotype value of the high phenotypic subpopulation increased sequentially from the first to the third population pair. Correspondingly, the average phenotype value of low phenotypic subpopulation decreased sequentially. As expected, the average phenotypic value of population pairs for all five meat quality traits are significant differences (*p* < .01).

### Genomic Characters Among Phenotypic Gradient Differential Population Pairs

**FIGURE 2:**
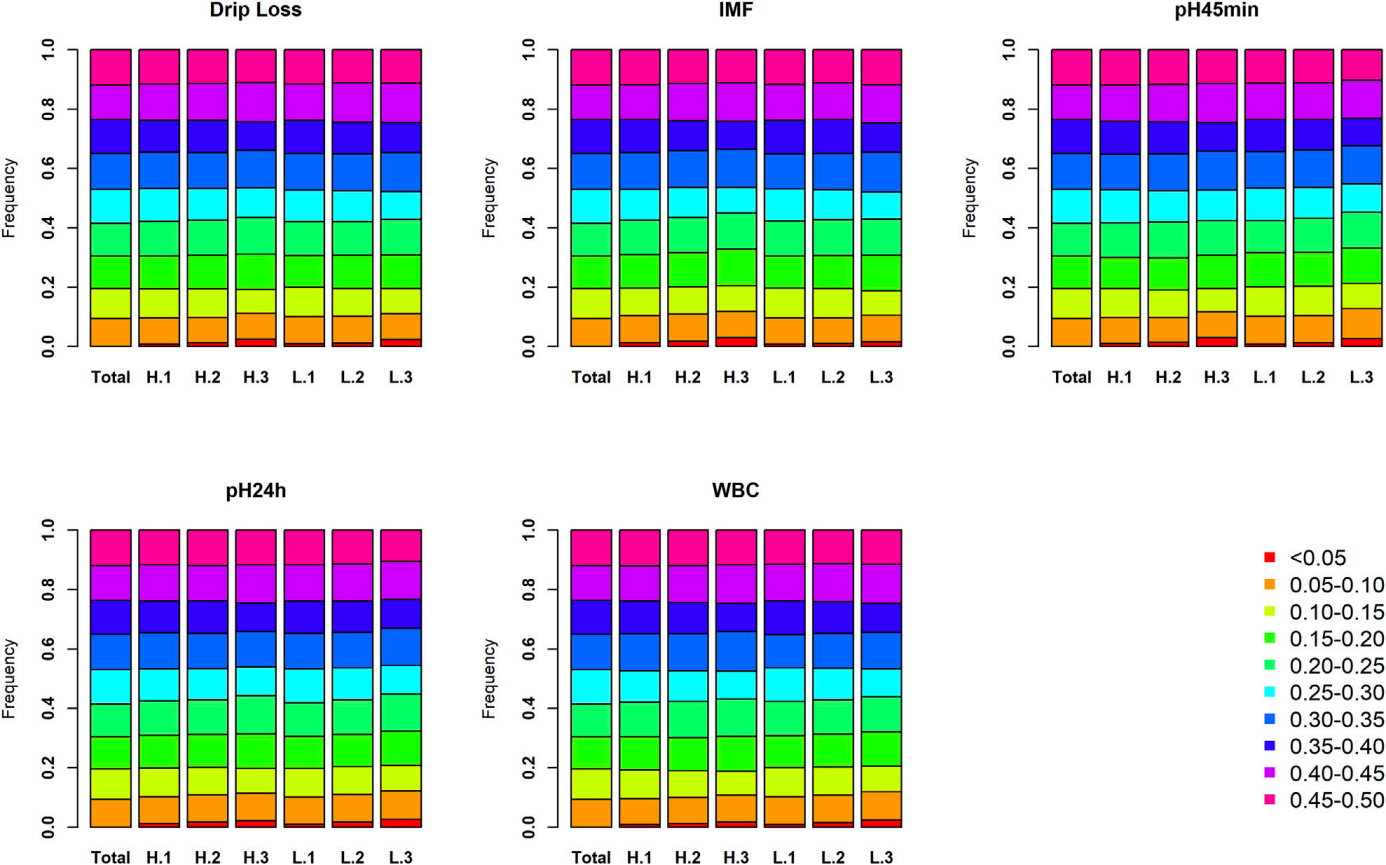
The proportion of each MAF bin in each subpopulation for five meat quality traits. The total represents the proportion of each MAF bin in source population. Similarly, H.3 (H.2, H.1) and L.3 (L.2, L.1) represent the proportion of each MAF bin in the two subpopulations of third (second and first) population pair.

To explore the influence of population division in this analysis, minor allele frequency, and linkage disequilibrium were investigated among different populations. As expected, the distribution of the minor allele frequency (MAF) in each subpopulation is similar to the results using all 233 large white pigs (Figure 2). The proportion of MAF between 0 and 0.1 increases slightly as the sample size of each subpopulation decreases. Simultaneously, the trend of linkage disequilibrium decay is similar in all subpopulations (Supplementary Figure S1). The results show that the genomic characters of each subpopulation in the three phenotypic gradient population pairs have little difference. Therefore, the population division would not affect the identification of selection signatures. Further PCA analysis indicated that the population division for each trait will not cause population stratification (Supplementary Figures S2–S6). Overall, the results can prove that the trait-specific selection signatures revealed in this study are caused by phenotypic differences rather than an accidental genomic structural difference because of population division.

### Trait-Specific Selection Signatures

**FIGURE 3:**
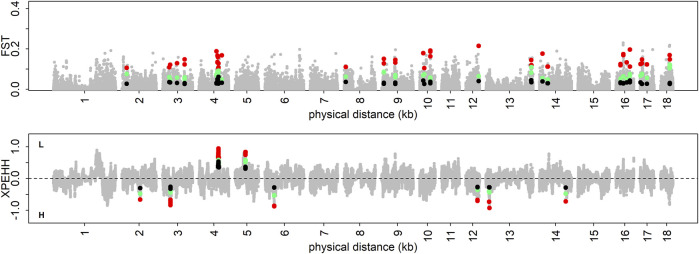
Visualization of trait-specific selection signatures for DL. The colored dots represent trait-specific selection signatures.

Based on the constructed population pairs with extreme differences in phenotypes, XPEHH and F_ST_ were employed to identify positive selection signatures. In this analysis, about 370 positive selection signatures were detected by each method in each population pair for each trait (Supplementary Table S2). Then, these positive selection signatures with gradient changes in three phenotypic gradient differential population pairs were defined as trait-specific selection signatures. Finally, 55, 49, 43, 111, and 49 trait-specific selection signatures were detected in DL, IMF, pH45min, pH24h, and WBC using F_ST_ test, respectively. Similarly, the XPEHH test revealed 59, 102, 159, 43, and 64 trait-specific selection signatures in DL, IMF, pH45min, pH24h, and WBC, respectively (Figure 3, Supplementary Figures S7–S10).

### Candidate Genes Overlapping With Trait-Specific Selection Signatures

Based on the trait-specific selection signatures, the genes overlapping with the potential selection regions were determined using the pig reference genome (Sscrofa11.1). Enrichment analysis showed that no significant biological terms may be associated with five meat quality traits after multiple correction in this study (Supplementary Table S3). Nevertheless, we note that genes harboring trait-specific selection signatures are related with muscle morphology by functional annotations based on the MGI database (Table 1). For example, the *SCYL3* gene that overlaps with the DL-specific selection signatures is related to abnormal morphology of mouse skeletal muscle fibers through orthologous alignment and MGI annotation (Dickinson et al., 2016). *ENSSSCG00000027613* genes were found overlapping with the IMF-specific selection signature. Note that the ortholog gene of *ENSSSCG00000027613* is *Trdn* in the mouse, which is related to abnormal skeletal muscle fiber triad morphology (Oddoux et al., 2009). The previous study indicates that this gene plays an important role in skeletal muscle function and structure. In addition, the ortholog gene *FBX O 32* and *CASQ1* are separately related to reducing the susceptibility to induced muscle atrophy and abnormal muscle physiology (Mosca et al., 2016; Singh et al., 2017). Both of them were identified as a potential selection signatures in the IMF content trait.

**TABLE 1:** The summary of functional annotation of the trait-specific selection signatures.

Chr	Position (bp)^a^	XPEHH (F_ST_) Scores^b^	Genes^c^	Trait	MGI phenotype
10	20392326–20707474	(0.03 < 0.07 < 0.11)	*DENND1B*	-,DL	MP:0001257_increased body length
12	51773420–51794447	−0.26 > −0.35 > −0.67	*SCIMP*	High, DL	MP:0001260_increased body weight
4	81133591–81176676	(0.05 < 0.07 < 0.13)	*SCYL3*	-,DL	MP:0003084_abnormal skeletal muscle fiber morphology
5	46244283–46274049	0.31 < 0.52 < 0.75	*ENSSSCG000000005*(*Smco2*)	Low, DL	MP:0003960_increased lean body mass
1	39180526–39339224	(0.06 < 0.09 < 0.12)	*ENSSSCG0000002761*(*TRDN*)	-,IMF	MP:0009411_abnormal skeletal muscle fiber triad morphology
10	25822287–25826726	0.30 < 0.41 < 0.55	*ENSSSCG00000010936*(*Fabp3*)	Low, IMF	MP:0002118_abnormal lipid homeostasis
10	25707711–25735067	0.30 < 0.42 < 0.55	*ZNF367*(*Zfp367*)	Low, IMF	MP:0005553_increased circulating creatinine level
4	15895092–15930934	(0.04 > 0.09 > 0.13)	*FBXO32*	-, IMF	MP:0004064_decreased susceptibility to induced muscular atrophy
4	90275213–90294819	−0.38>−0.46>−0.66	*CASQ1*	High, IMF	MP:0002106_abnormal muscle physiology
7	25751185–25808637	0.31 < 0.44 < 0.64	*GFRAL*	Low, IMF	MP:0001259_abnormal body weight
11	23237692–23894699	(0.05 < 0.08 < 0.14)	*ENOX1*	-,pH45m	MP:0000062_increased bone mineral density
11	68920906–69301332	0.53 < 0.62 < 0.96	*PCCA*	Low, pH45m	MP:0001429_dehydration
7	114035383–114541592	−0.41 < −0.48 < −0.55 (0.06 < 0.11 < 0.18)	*RIN3*	High, pH45m	MP:0005560_decreased circulating glucose level
3	24815010–24835836	0.33 < 0.36 < 0.55	*CRYM*	Low, pH24h	MP:0005472_abnormal triiodothyronine level
4	123674697–123779615	(0.05 < 0.08 < 0.12)	*FNBP1L*	-,pH24h	MP:0003961_decreased lean body mass
1	268581835–268590836	−0.35 > −0.46 > −0.55 (0.04 < 0.07 < 0.11)	*PTGES2*	High, WBC	MP:0001785_edema
8	132035777–132200420	(0.03 < 0.06 < 0.08)	*PTPN13*	-,WBC	MP:0001261_obese

a This column is the position of candidate genes.

b This column is the XPEHH(F_ST_) scores of three phenotypic gradient change population pairs.

c The gene in brackets is the mouse ortholog gene.

### A Highlighted IMF-specific Selection Signature Region in Chromosome 4

MF is one of the most important meat quality traits in pig breeding programs. Here, a promising genomic region (SSC4 93544042–95179724bp) that was detected by XPEHH and F_ST_ simultaneously is associated with high IMF content. Based on the pigQTLdb, we found that 10 QTLs related to meat quality and fat metabolism are harbored in this genomic region (Supplementary Table S4). Further bioinformatics annotation in this region found that *USF1*, *NDUFS2*, *PIGM*, *IGSF8*, and *CASQ1* gene can be considered as functional candidate genes for meat quality. Among them, the *USF1* gene plays an important role in lipid homeostasis; *PIGM* is related to lipid metabolism and catabolism; *IGSF8* and *CASQ1* are related to muscle development; *NDUFS2* is related to the synthesis of energy metabolism (Table 2) (Kim et al., 2015).

**TABLE 2:** The annotation of seven potential candidate genes in SSC4 (Kim et al., 2015).

Chr	Position (bp)^a^	XPEHH (F_ST_) Scores^b^	*Trait*	Genes	Function
4	89395156–89401258	−0.42 > −0.50 > −0.64 (0.04 < 0.06 < 0.15)	High, IMF	*USF1*	Lipid homeostasis
4	89247150–89262159	−0.42 > −0.50 > −0.64 (0.04 < 0.06 < 0.15)	High, IMF	*NDUFS2*	Integration of energy metabolism
4	90461734–90467492	−0.34 > −0.43 > −0.61	High, IMF	*PIGM*	Lipid metabolism and catabolism `
4	90370235–90405480	−0.34 > −0.44 > −0.64	High, IMF	*IGSF8*	Muscle development
4	90275213–90294819	−0.38 > −0.46 > −0.66	High, IMF	*CASQ1*	Muscle development

a This column is the position of candidate genes.

b This column is the XPEHH(F_ST_) scores of three phenotypic gradient change population pairs.

## Discussion

In this study, we planned to map some candidate genes of meat quality using population differentiation–based selection signature methods through constructing phenotypic gradient difference population pairs. In theory, meat quality traits are quantitative traits, controlled by minor-effect polygenes, and their genetic mechanisms are complex (Falconer and Mackay, 1960; Hill et al., 2007). Although these loci are related to the complex traits, they are not easy to detect through genome-wide association analysis because of their small effect, especially after multiple correlations. However, these loci have been shaped by human-driven selection; they can be detected through sweep analysis from the perspective of population genetics (Qanbari and Simianer 2014). Therefore, it is theoretically feasible to use population differentiation–based selection signature methods to identify trait-specific selection signatures through constructing phenotypic gradient population pairs. The results of the functional annotation also support this hypothesis. A series of genes and QTLs harboring trait-specific selection signatures are related with meat quality traits (Table 1, Supplementary Table S4). There is no doubt that this strategy also has a shortcoming that the fixed loci caused by selection will not be identified after MAF quality control. In addition, both the sample size of the third population pair and the gradient across different population pairs are at least 40 unrelated individuals according to our previous study (Ma et al., 2015). Although small sample sizes appear to be sufficient in the detection of selection signatures according to simulation research, there is no doubt a larger sample size will contribute to decrease the risk of detection bias.

The genetic mechanism of meat quality traits is complex. The candidate genes related to meat quality traits discovered in this study may have pleiotropism effects. As shown in Table 1, some orthologous genes, such as *SCIMP*, *ENSSSCG00000000550*, *GFRAL*, and *FNBP1L*, are related to the body weight and lean body weight of mice (George et al., 2014; Tsai et al., 2019). We found that some genes overlapping with meat quality traits–specific selection signatures are also associated with lipid traits, such as *ENSSSCG00000010936* and *PTPN13*. Simultaneously, the orthologous gene of *ZNF367* that was considered an IMF-specific selection signature is related to the increase in circulating levels of creatinine in mice, which is consistent with the fact that creatinine has flavor properties. After the annotation of pigQTLdb, we note that trait-specific selection signals are mainly associated with QTLs for meat quality traits, but there are also QTLs related to backfat thickness, body weight, and body length (Supplementary Table S4). In general, there is a negative correlation between backfat thickness and meat quality traits, especially DL and IMF. This result indicates that the artificial selection of backfat thickness and body size in recent pig breeding programs should have a significant impact on meat quality traits.

Because the XPEHH method can identify the selected population, this study can further study the complex genetic mechanism of meat quality traits (Sabeti et al., 2007). In general, the artificial selection of economic traits shows a single direction of phenotypic changes, but the corresponding genetic basis changes are in two directions: increasing and decreasing effects. Taking IMF as an example, 48 and 54 selection signatures were separately detected in low and high IMF subpopulations (Supplementary Table S5). Note that the biological phenotypes of the *CASQ1* and *ENSSSCG00000010936* genes identified in the high and low IMF subpopulations are abnormal muscle physiology and abnormal lipid homeostasis based on MGI annotation (Dickinson et al., 2016). In addition, 115 trait-specific selection signatures detected in the low pH45min subpopulation are more than 44 trait-specific selection signatures detected in the high pH45min subpopulation. This is consistent with the fact that the breeding direction of lean pigs under artificial selection leads to a decrease in water holding capacity and a rapid decrease in muscle pH after slaughter in recent years. However, 37 trait-specific selection signatures detected in the low DL subpopulation are more than 22 trait-specific selection signatures detected in the high DL subpopulation (Supplementary Table S5). This further indicates the complexity of the genetic mechanism of meat quality traits. Meat quality traits are affected by many factors, including fat metabolism, transportation, muscle fiber formation, and physiological conditions. Therefore, it can be inferred that further multi-omics integrated analysis is an important way to analyze the genetic mechanism of meat quality traits in the future.

## Conclusion

In this study, we propose a new strategy to identify trait-specific selection signatures. The application in five meat quality traits in large white pigs indicate that this strategy is promising in gene mapping. Furthermore, we detected a series of genomic selection signatures and identified some genes related to meat quality traits, such as *USF1*, *NDUFS2*, *PIGM*, *IGSF8*, and *CASQ1*, which provide a reference for future pig breeding.

## Data Availability

The original contributions presented in the study are included in the article/Supplementary Materials, further inquiries can be directed to the corresponding author.
