# Supplementary material for: Trait-specific Selection Signature Detection Reveals Novel Loci of Meat Quality in Large White Pigs
Source: Front Genet. 2021 Nov 16;12:761252. doi: 10.3389/fgene.2021.761252 (PMC8635012; doi:10.3389/fgene.2021.761252)
Supplement: Supplementary file 1 [file DataSheet1.zip › Suppl. Tables & Figures.DOC]

**Trait-specific Selection Signatures Detection Reveals Novel Loci of Meat Quality in** **Large White pigs**

Yu Shen^#^, Haiyan Wang^#^, Jiahao Xie, Zixuan Wang, Yunlong Ma*

Key Laboratory of Agricultural Animal Genetics, Breeding, and Reproduction of the Ministry of Education & Key Laboratory of Swine Genetics and Breeding of the Ministry of Agriculture, Huazhong Agricultural University, Wuhan 430070, China

***Correspondence and requests for materials should be addressed to:** Yunlong Ma (Yunlong.Ma@mail.hzau.edu.cn)

**# These authors contributed equally to this work.**

**Office mailing address:** College of Animal Sciences & Technology, Huazhong Agricultural University, No. 1, Shizishan Street, Hongshan District, Wuhan 430070, People’s Republic of China
Tel: +86 02787282091

**Table S1 the correlation coefficients and the other descriptive statistics of five meat quality traits**

|  | **DL** | **IMF** | **pH45m** | **pH24h** | **WBC** |
| --- | --- | --- | --- | --- | --- |
| **DL** | 1.34±0.47% | 0.04 | 0.01 | 0.002 | <0.001 |
| **IMF** | 0.15 | 2.25±0.50% | 0.41 | 0.08 | 0.02 |
| **pH45m** | 0.20 | -0.06 | 6.60±0.23 | 0.004 | 0.69 |
| **pH24h** | 0.23 | 0.13 | 0.22 | 5.56±0.18 | <0.001 |
| **WBC** | 0.30 | 0.17 | -0.03 | 0.29 | 4.06±0.55% |

Note: The lower and upper triangle is the correlation coefficient and its p-value, respectively. The diagonal is the mean and standard deviation of all individual phenotypes of the Large White pigs.

**Table S2 the summary of significant selection signatures for five meat quality traits.**

|  |  | **DL** | **IMF** | **pH45min** | **pH24h** | **WBC** |
| --- | --- | --- | --- | --- | --- | --- |
| **1st population pair** | XPEHH;  F_ST_ | 369;  370 | 370;  370 | 370;  371 | 372;  373 | 370;  370 |
| **2nd population pair** | XPEHH;  F_ST_ | 369;  378 | 370;  370 | 370;  374 | 372;  372 | 370;  370 |
| **3rd population pair** | XPEHH;  F_ST_ | 369;  373 | 370;  372 | 370;  379 | 370;  374 | 370;  378 |
| **Common Selection Signatures** | XPEHH;  F_ST_ | 65;  56 | 128;  50 | 173;  43 | 48;  111 | 79;  51 |
| **Trait-specific Selection Signatures** | XPEHH;  F_ST_ | 59;  55 | 102;  49 | 159;  43 | 43;  111 | 64;  49 |

**Table S3 the results of GO analysis and pathway analysis.**

See Supplementary Table S3.xlsx

**Table S4 QTLs overlapped with the trait-specific selection signatures identified by F_ST_ and XPEHH simultaneously.**

| **Chr.** | **Position(bp)** | **XPEHH (F_ST_) Scores** | **Trait, Direction** | **QTL Name** |
| --- | --- | --- | --- | --- |
| 1 | 105410407-106239216 | 0.38<0.41<0.52  (0.04<0.05<0.08) | WBC, Low | Mean corpuscular hemoglobin content QTL；Mean corpuscular volume QTL |
| 1 | 268398928-269207079 | -0.35>-0.46>-0.55  (0.04<0.07<0.11) | WBC, High | Time in feeder per visit QTL；Total number born alive QTL；Drip loss QTL |
| 3 | 108199147-108626470 | -0.37>-0.43>-0.56 (0.10<0.12<0.20) | PH24h, High | Meat color-L QTL；LDL cholesterol QTL |
| 4 | 84273030-86251706 | 0.37<0.39<0.66 (0.03<0.08<0.11) | DL, Low | Days to 110 kg QTL；Meat color a* QTL；Shoulder subcutaneous fat thickness QTL；Drip loss QTL；  Body weight (weaning) QTL； |
| 4 | 89139474-89869667 | -0.42>-0.50>-0.64 (0.04<0.06<0.15) | IMF, High | Feed conversion ratio QTL |
| 4 | 93544042-95179724 | 0.38<0.68<0.98 (0.04<0.09<0.16) | pH45m, Low | Double-bond index QTL；Oleic acid content QTL；Polyunsaturated fatty acid to saturated fatty acid ratio QTL；  Bone/lean meat ratio in ham QTL；Meat color a* QTL；Palmitoleic acid content QTL；Linoleic acid content QTL；Palmitic acid content QTL；Vaccenic acid to palmitoleic acid ratio QTL；Average daily gain QTL； |
| 4 | 114429350-116073400 | -0.42>-0.50>-0.57 (0.05<0.07<0.18) | pH24h, High | Feed conversion ratio QTL；Scapula length QTL；Age at puberty QTL；Teat number QTL；Meat color b* QTL；  Spinal curvature QTL；Thoracic vertebra number QTL； |
| 6 | 93935777-94423271 | -0.30>-0.39>-0.54 (0.05<0.12<0.18) | pH45m, High | Feed conversion ratio QTL；Lumbar vertebra number QTL；Average daily gain QTL；Average backfat thickness QTL；Backfat at first rib QTL；Backfat between 3rd and 4th last ribs QTL；Belly weight QTL；Rib weight QTL；Intramuscular fat content QTL；Body weight (21 weeks) QTL；Body weight (25 weeks) QTL；Carcass weight (hot) QTL；Shoulder weight QTL；Loin weight QTL；Loin muscle area QTL；Linoleic acid content QTL；Eicosenoic acid content QTL；Saturated fatty acid content QTL；Polyunsaturated fatty acid content QTL；Body weight QTL；Thoracolumbar vertebra number QTL；Lumbar vertebra number QTL；Chest width QTL； |
| 6 | 150965116-151486680 | 0.33<0.41<0.57  (0.07<0.09<0.15) | pH24h, Low | Rump width QTL；Litter size QTL； |
| 7 | 104204027-104725604 | -0.35>-0.47>-0.55 (0.05<0.12<0.17) | pH45m, High | Mean corpuscular volume QTL；Intramuscular fat content QTL；Number of ribs QTL；Average daily gain QTL；Teat number QTL； |
| 7 | 110888111-111628030 | -0.337>-0.34>-0.65 (0.09<0.13<0.21) | pH45m, High | Coping behavior QTL |
| 7 | 114127837-114609265 | -0.41>-0.48>-0.55 (0.06<0.11<0.18) | pH45m, High | Litter size QTL；Ham weight QTL； |
| 10 | 25360313-26889225 | 0.30<0.42<0.55 (0.05<0.10<0.11) | IMF, Low | Maternal infanticide QTL； |
| 11 | 67647917-70751880 | 0.51<0.57<1.13 (0.08<0.10<0.21) | pH45m, Low | Lean meat percentage QTL；pH 45 minutes post mortem QTL；Backfat at rump QTL；Litter size QTL； |
| 14 | 77923861-78439289 | -0.37>-0.52>-0.60 (0.06<0.07<0.20) | IMF, High | Lymphocyte number QTL；Spinal curvature QTL；Thoracic vertebra number QTL； |
| 14 | 131157261-131589979 | -0.27>-0.34>-0.53  (0.04<0.05<0.10) | WBC, High |  |
| 16 | 76053300-76453300 | 0.33<0.60<0.63 (0.04<0.07<0.13) | IMF, Low | Average feeding rate QTL；Hind leg conformation QTL；Average backfat thickness QTL |

**Table S5 the summary of XPEHH scores for trait-specific selection signatures.**

|  | **DL** | **IMF** | **pH45min** | **pH24h** | **WBC** |
| --- | --- | --- | --- | --- | --- |
| **Low phenotypic value in subpopulation (+)** | 37 | 48 | 115 | 15 | 28 |
| **High phenotypic value in subpopulation (-)** | 22 | 54 | 44 | 28 | 36 |

Note: “+” and “-” indicated that selection signatures were detected in the corresponding subpopulations, respectively.

**Figure S1** The decay of linkage disequilibrium of all subpopulations across the genome. The pairwise linkage disequilibrium (r2) is plotted against the corresponding physical distances. High_1 (High_2, High_3) and Low_1 (Low_2, Low_3) represent the two subpopulations of 1st (2nd and 3rd) population pair. LD: linkage disequilibrium.

**Figure S2** Principal component analysis of Large White pigs. The colored dots indicate the extreme individuals of three population pairs for drip loss. H_1 (H_2, H_3) and L_1 (L_2, L_3) represent the two subpopulations of 1st (2nd and 3rd) population pair.

**Figure S3** Principal component analysis of Large White pigs. The colored dots indicate the extreme individuals of three population pairs for IMF. H_1 (H_2, H_3) and L_1 (L_2, L_3) represent the two subpopulations of 1st (2nd and 3rd) population pair.

**Figure S4** Principal component analysis of Large White pigs. The colored dots indicate the extreme individuals of three population pairs for pH24h. H_1 (H_2, H_3) and L_1 (L_2, L_3) represent the two subpopulations of 1st (2nd and 3rd) population pair.

**Figure S5** Principal component analysis of Large White pigs. The colored dots indicate the extreme individuals of three population pairs for pH45min. H_1 (H_2, H_3) and L_1 (L_2, L_3) represent the two subpopulations of 1st (2nd and 3rd) population pair.

**Figure S6** Principal component analysis of Large White pigs. The colored dots indicate the extreme individuals of three population pairs for WBC. H_1 (H_2, H_3) and L_1 (L_2, L_3) represent the two subpopulations of 1st (2nd and 3rd) population pair.

**Figure S7** Visualization of trait-specific selection signatures for intramuscular fat (IMF). The colored dots represent trait-specific selection signatures.

**Figure S8** Visualization of trait-specific selection signatures for pH at 45 min (pH45min). The colored dots represent trait-specific selection signatures.

**Figure S9** Visualization of trait-specific selection signatures for pH at 24 hours (pH24h). The colored dots represent trait-specific selection signatures.

**Figure S10** Visualization of trait-specific selection signatures for water binding capacity (WBC). The colored dots represent trait-specific selection signatures.
